# Complete Genome Sequence of the Photoautotrophic and Bacteriochlorophyll *e*-Synthesizing Green Sulfur Bacterium *Chlorobaculum limnaeum* DSM 1677^T^

**DOI:** 10.1128/genomeA.00529-17

**Published:** 2017-06-15

**Authors:** Marcus Tank, Zhenfeng Liu, Niels-Ulrik Frigaard, Lynn P. Tomsho, Stephan C. Schuster, Donald A. Bryant

**Affiliations:** a Department of Biochemistry and Molecular Biology, Eberly College of Science, The Pennsylvania State University, University Park, Pennsylvania, USA; b Department of Biology, University of Copenhagen, Helsingør, Denmark; c Department of Chemistry and Biochemistry, Montana State University, Bozeman, Montana, USA

## Abstract

*Chlorobaculum limnaeum* DSM 1677^T^ is a mesophilic, brown-colored, chlorophototrophic green sulfur bacterium that produces bacteriochlorophyll *e* and the carotenoid isorenieratene as major pigments. This bacterium serves as a model organism in molecular research on photosynthesis, sulfur metabolism, and bacteriochlorophyll biosynthesis. We report here the complete genome sequence.

## GENOME ANNOUNCEMENT

*Chlorobaculum limnaeum* DSM 1677^T^ is a brown-colored, chlorophototrophic green sulfur bacterium (GSB) that belongs to the family *Chlorobiaceae* within the phylum *Chlorobi* (1, 2). It was originally isolated from Lake Kinneret, a monomictic, freshwater lake in Israel (https://www.dsmz.de/catalogues/details/culture/DSM-1677.html). Like all other members of the *Chlorobiaceae, C. limnaeum* is an anaerobic, anoxygenic chlorophotoautotroph, and all known required genes for this capability were detected in the genome (3). Light energy is harvested by chlorosomes, which are also found in the phototrophic *Chloroflexaceae* (4), the recently discovered *Chloracidobacterium thermophilum* (5), and “*Candidatus* Thermochlorobacter aerophilum” (2). Sulfide is the preferred electron donor, and CO_2_ fixation occurs through the reverse tricarboxylic acid cycle (3). GSB that are green in color synthesize chlorosomes containing bacteriochlorophyll (BChl) *c* or/and BChl *d* and the carotenoid chlorobactene, but brown-colored GSB produce chlorosomes containing BChl *e* and, usually, the carotenoid isorenieratene (3).

In contrast to the thermophilic, BChl *c*-containing, and naturally transformable *Chlorobaculum tepidum* TLS^T^, *C. limnaeum* only recently became a model organism for studies on the evolution of photosynthesis, sulfur metabolism, and biosynthesis of BChls and carotenoids in brown-colored GSB. For example, the genomic data for *C. limnaeum* helped produce a mutant with chlorosomes containing only BChl *f* by inactivation of the *bchU* gene (6). BChl *f* is C-20 demethyl Bchl *e* and is analogous to the C-20 demethyl BChl *c*, namely, BChl *d*, which is found in green-colored GSB (6). Another recent study demonstrated that BciD of *C. limnaeum* is a radical *S*-adenosyl-l-methionine enzyme that converts the C-7 methyl group of bacteriochlorophyllide *c* or *d* into the formyl group of bacteriochlorophyllide *e* or *f*, respectively (7).

The closed genome of *C. limnaeum* DSM 1677^T^ consists of a single chromosome with 2,797,276 bp and a G+C content of 56.4 mol%; the genome is comparable in size to those of other GSB genomes (2.0 to 3.1 Mbp) (3). No plasmids were detected. Annotation using the NCBI Prokaryotic Genome Annotation Pipeline (https://www.ncbi.nlm.nih.gov/genome/annotation_prok/) predicted 2,452 protein-coding genes, 49 genes encoding tRNAs, and 2 rRNA operons with identical 5S, 16S, and 23S nucleotide sequences.

*C. limnaeum* DSM 1677^T^ was grown under anoxic conditions at ~25°C in CL^-^ medium (8) at ~50 µmol photons m^-2^ s^-1^. Purified genomic DNA was sequenced on a 454 pyrosequencer (GS FLX+; Roche) to an average depth of >60×. Reads (1,281,523 reads; average length, 381 ± 123 bp) were assembled using the Newbler assembler (Roche) into 89 contigs of at least 500 bp. The contigs were compared to the genome of *C. tepidum* (9) using PGA (10) to predict the arrangement of and the connections between the contigs. PCR amplification and Sanger sequencing were used for gap closure. The final assembly was carried out using the phred/phrap/consed software package.

### Accession number(s).

The genome sequence has been deposited at DDBJ/EMBL/GenBank under the nucleotide sequence with accession number CP017305.
